# Atypical Causes of Urinary Tract Obstruction

**DOI:** 10.1155/2019/4903693

**Published:** 2019-02-27

**Authors:** Ernie Yap, Moro Salifu, Tahir Ahmad, A. Sanusi, Anthony Joseph, Mary Mallappallil

**Affiliations:** State University of New York, Downstate Medical Center, Brooklyn, NY 11203, USA

## Abstract

Acute kidney injury due to urinary tract obstruction invariably suggests lower urinary tract obstruction or bilateral ureteric obstruction since obstruction of a single kidney while the contralateral kidney is normal and not obstructed would not cause a perceptible rise in creatinine. Assuming a total body volume of 42 L, 70 kg male that generates approximately 1400 mg of creatinine daily (20 mg/kg/day) who has complete urinary tract obstruction would experience a 3.33 mg/dL per day increase in serum creatinine. Thus, for an individual who had prior normal renal function and who presents with a creatinine of 30 mg/dL, one could surmise that the obstructive pathology had lasted at least 10 days. However, the rise in serum creatinine is a poor marker of renal injury and subsequent prognosis. Urinary tract obstruction leading to AKI can be due to a variety of causes, and its management is tailored to the underlying etiology. This case series describes the varied clinical course of four patients at our center who experienced AKI from atypical causes of obstructive uropathy. Current and future diagnostic modalities and caveats in the treatment of this disease entity are also discussed.

## 1. Introduction

Acute kidney injury (AKI) is one of the commonest reasons for nephrology consultation. Of these, 5-10% of cases are due to urinary tract obstruction (UTO). UTO may result from stones or external compression from tumors, adenopathy, or retroperitoneal fibrosis. Early diagnosis of UTO is important since most cases can be corrected and delay in therapy can lead to irreversible renal injury. The degree of azotemia at time of presentation does not reflect the extent of kidney injury and subsequent prognosis. Herein we present a series of four cases of obstructive uropathy that had various atypical causes and natural histories.

## 2. Patient 1. Multi-Pronged Assault by Plasma Cell Dyscrasias

A 40-year-old man of West African origin was diagnosed with IgG lambda multiple myeloma with urinary free lambda light chains of 0.34 mg/L (normal range: 0.24-6.66 mg/L)) and plasmacytomas involving the pleura and bone marrow. He was treated with bortezomib-based chemotherapy with no response. Salvage chemotherapy resulted in tumor lysis syndrome (K 7.4 mmol/L; uric acid 12.9 mg/dL, serum creatinine 2.0 mg/dL) that resolved with chemotherapy de-escalation, rasburicase, and a single hemodialysis session. His renal function then returned to normal and the patient continued salvage chemotherapy as an outpatient. Eight months later, he presented to the Emergency Department (ED) with abdominal discomfort and AKI (serum creatinine 18.2 mg/dL) and was found to have bilateral severe hydronephrosis, due to upper ureteral obstruction caused by retroperitoneal adenopathy. Upon relief with percutaneous nephrostomy, his renal function normalized (serum creatinine 1.1 mg/dL), with suppression of monoclonal protein (M-spike 1.4 g/dL). Two months later, the patient was admitted to hospital for another episode of AKI that progressed despite supportive care. His M-spike was 5.3 g/dL and urinary free lambda light chain was 5080 mg/dL, consistent with myeloma kidney. He ultimately required hemodialysis support. The patient succumbed to complications of multiple myeloma and sepsis soon after, 14 months after diagnosis.

## 3. Patient 2. Extreme Elevation of Serum Creatinine with Prostate Adenocarcinoma from Urethral Obstruction with Full Recovery of Renal Function

A 66-year-old man presented to the ED for a 3-week history of bilateral leg edema, distended abdomen, dysuria, and anorexia. He was recently treated for a urinary tract infection. Physical examination disclosed a distended bladder. Initial laboratory data revealed AKI with hyperkalemia (K 7.9 mEq/L, blood urea nitrogen (BUN) was 148 mg/dL and serum creatinine of 35.92 mg/dL); an urgent nephrology consultation was requested for emergency hemodialysis. In the meantime, a bladder catheter was placed with approximately 9 liters of urine output. Over the next 30 hours the patient's laboratory data improved dramatically (Table 1). His BUN dropped to 36 mg/dL within one day. The cause of AKI was later found to be UTO secondary to prostatic adenocarcinoma. His renal function improved spontaneously without the need for hemodialysis.

## 4. Patient 3. Extreme Elevation of Serum Creatinine with Benign Prostatic Hypertrophy with Full Recovery of Renal Function

A 58-year-old man with mild mental retardation and diabetes mellitus was brought to the ED for vomiting, diarrhea, and progressive weakness for several days. He had significant azotemia with serum creatinine of 31 mg/dL and BUN of 187 mg/dL with hyperkalemia and high anion gap metabolic acidosis (Table 1). Computed tomography (CT) of the abdomen showed moderate to severe bilateral hydronephrosis and a very large prostate gland protruding into the bladder, which is significantly distended. Renal ultrasonography showed mild bilateral hydronephrosis. The patient was treated medically for hyperkalemia, and a Foley catheter was placed with resultant good urinary flow, followed by rapid resolution of his azotemia and electrolyte derangements. His BUN decreased to 41 mg/dL in 2 days. A prostate biopsy performed later revealed benign prostatic hyperplasia.

## 5. Patient 4. Endometriosis Presenting as an Intraluminal Colon Mass Causing Chronic Ureteral Obstruction

A 40-year-old woman with a past medical history of hypertension and severe endometriosis presented to the ED with a serum creatinine of 17 mg/dL and BUN of 86 mg/dL. Renal sonogram disclosed moderate hydronephrosis with hydroureter compressed by a large myomatous uterus. A hysterectomy was performed which resulted in renal function improvement but did not normalize. However, the patient continued to develop multiple bouts of AKI and progressive chronic kidney disease (CKD) for the next several years. An episode of lower gastrointestinal bleeding prompted a colonoscopy that revealed a mass at the rectosigmoid junction which was resected and confirmed to be colonic endometriosis and leuprolide was started. By this time, the patient's CKD had progressed to end-stage renal disease and she was placed on maintenance hemodialysis.

## 6. Discussion

We present these four cases to illustrate atypical causes of UTO leading to AKI. A thorough history-taking and physical examination is imperative in the approach of a patient with AKI from any cause. Other nonobstructive causes of AKI such as ischemia, tubulointerstitial injury, glomerular diseases, tubular nephritis, or CKD from systemic illnesses ought to be considered as part of the differential diagnosis.

There are no pathognomonic findings attributed to UTO-related AKI. Palpation of the abdomen may reveal a suprapubic mass that typically represents a distended bladder. If UTO is suspected, the initial imaging modality of choice is ultrasonography, which has a high sensitivity and specificity even when performed and interpreted by nonradiologists [1]. It does not require radiocontrast and the detection of hydronephrosis is a sensitive marker for urinary tract obstruction [2–4]. In addition, ultrasonography can determine the size and shape of the kidneys. However, it is noteworthy that sonographic demonstration of significant renal parenchymal loss does not necessarily predict an irreversible cause for renal failure [2]. Patient 3 has renal sonographic findings that were discordant with clinical features and CT imaging. Renal ultrasonography can be negative in early obstruction, when the pyelocalyceal system is not yet dilated [5]. In such cases, CT scanning is useful. Also, scintigraphy with use of technetium 99m labeled mercaptoacetyltriglycine (MAG3) or diethylenetriaminepentaacetic acid (DTPA) can evaluate renal perfusion, glomerular function, and gross renal structure as well as excretory function.

Following relief of UTO, there is usually a resultant increase in sodium and water excretion even with the temporary decrease in glomerular filtration rate, as exemplified by Patient 2. This is because the volume expansion that occurs during UTO suppresses antidiuretic hormone (ADH) and aldosterone, leading to minimal reabsorption at the distal and collecting duct level until volume depletion sets in to re-establish reabsorption. It is usually self-limiting and can be a composite of both solute (urea and sodium) and water diuresis. Nevertheless, some patients will continue to eliminate salt and water even after homeostasis has been reached [6], a condition referred to as pathologic postobstructive diuresis (POD). These patients are at risk of severe dehydration, electrolyte imbalances, hypovolemic shock, and even death if not recognized and treated promptly. Patients with pathologic POD require strict monitoring of fluid status and serum electrolyte levels. Intravenous fluid replacement is often warranted and the type and amount of fluids should be tailored to the patient's serum electrolyte levels and clinical hydration status.

Pathologic POD is estimated to occur in 0.5% to 52% of patients who underwent relief of obstruction [7]. Animal experiments have shown that, in POD, solute and water dynamics alone are inadequate to fully account for the natriuresis and diuresis that follows obstruction relief [8]. Using micro puncture and microinjection methods, McDougal et al. demonstrated that there are defects in both proximal and distal sodium reabsorption that persist for 2-3 days after obstruction relief [9]. In addition, the decrease in water reabsorption in POD has been postulated to be due to a decrease in sodium chloride (NaCl) gradient in the thin ascending loop of Henle. This in turn is brought on by decreased NaCl reabsorption in the medullary ascending limb, caused by increased prostaglandin synthesis. Prostaglandins increase blood flow within the vasa recta, thus facilitating solute washout from the medulla, diluting the countercurrent effect. The increased medullary prostaglandins also antagonize the effect of ADH, thereby reducing the water permeability of the collecting tubule, contributing to the decrease in urine osmolality [10].

Another important cause of morbidity in the patient whose UTO has been relieved is rapid lowering of plasma osmolality, leading to rapid water transfer into the central nervous system, resulting in cerebral edema. Symptoms of this disequilibrium syndrome can range from lethargy, headache, drowsiness, and confusion to serious complications such as seizures, cardiac arrhythmias, coma, and pulmonary edema [11] and are well described in patients undergoing hemodialysis for the first time but have been reported in nondialysis patients as well [12]. In our series, Patient 2 and Patient 3 demonstrated rapid improvement in BUN but did not develop any neurological sequelae. Risk factors for development of disequilibrium syndrome include markedly elevated BUN, concurrent CKD, hyponatremia, hepatic encephalopathy, malignant hypertension, pediatric age, or conditions that compromise the blood-brain barrier such as sepsis or thrombotic thrombocytopenic purpura [13].

Our experience with Patient 4 is a rare but growing body of literature describing an unusual presentation of UTO [14–17]. The ureters are rarely involved in endometriosis; however, the close anatomical proximity of the distal ureter to the female reproductive organs makes it a plausible target for the development of UTO. In addition to external compression, intrinsic renal involvement is also a consideration in the evaluation of a renal mass in a patient without traditional risk factors for malignancy, as a biopsy that confirms endometriosis may obviate an unnecessary nephrectomy [18].

The biochemical findings of AKI due to UTO are indistinguishable from AKI from other causes. The rapid normalization of serum creatinine upon obstruction relief shows that serum creatinine is poor marker of severity of injury. The observation that certain proteins are differentially expressed in nephropathy from obstructive causes and can be measured in whole urine and in urine exosomes suggests that more definitive biomarkers for UTO-related AKI such as KIM-1 may be feasible [19–22].

Battle et al. studied 13 patients with UTO with resultant AKI and found that they were associated with hyperkalemic hyperchloremic metabolic acidosis with features of impaired urinary acidification and mineralocorticoid deficiency [23]. This phenomenon was observed in most of our patients (Table 1) and we agree that the otherwise asymptomatic patient who presents with AKI and hyperkalemic hyperchloremic metabolic acidosis should undergo evaluation for UTO.

The treatment of relieving obstruction includes percutaneous nephrostomy, an invasive procedure that carries bleeding risk. It can be argued that this risk is augmented in patients with AKI, although this has not been clearly demonstrated. Desmopressin (1-deamino-8-D-arginine vasopressin, DDAVP) is a synthetic analogue of the antidiuretic hormone vasopressin that increases plasma concentrations of von Willebrand factor and factor VIII, which underlies its hemostatic effect [24]. Kim et al. prospectively investigated the potential of desmopressin to improve platelet dysfunction and lower bleeding risk after emergent invasive procedures in 23 patients who were uremic and who were infused with DDAVP before their invasive procedures (7 of which are percutaneous nephrostomy) and found that collagen/epinephrine-closure time was significantly shortened from 252.7±40.7 to 144.6±51.0 s with no severe bleeding event observed [25]. Also, DDAVP is used to treat diabetes insipidus due to its antidiuretic effect and has been used successfully to ameliorate pathologic POD [26].

*A Pertinent Question That Confronts Clinicians Is Does This Patient with UTO-AKI Require Hemodialysis?* The roles of hemodialysis in UTO-AKI are no different from AKI from other causes: correction of electrolyte derangements, acidosis, and fluid management. Our cases above illustrate that recovery of renal function invariably occurs as soon prompt corrective measures were undertaken, emphasizing the need for a systematic and global approach to the patient with unexplained AKI. However, prognosis is variable and some patients may progress to chronic kidney disease even after obstruction has been relieved, as interstitial fibrosis and inflammation may have supervened. The prolonged recovery seen in Patients 1 and 3 was associated with concomitant infection that resulted in acute tubular necrosis. Once a diagnosis is confirmed, it is sometimes not fully reversible such as malignancy and discussion of treatments including percutaneous nephrostomies, ureteral stents, resection of malignancy, or hemodialysis which should be undertaken with the goal of providing comfort and improving quality of life.

In summary, we have described a series of patients with UTO-related AKI that presented with significantly elevated creatinine, most of whom regained renal function after relief of obstruction without requiring urgent hemodialysis. There is an unmet medical need for the development of more specific biomarkers of AKI from UTO. The utility of DDAVP in patients with AKI undergoing invasive procedures merits further study.

## Figures and Tables

**Table 1:** Characteristics of patients with UTO.

Patient #	1	2	3	4
Peak creatinine (mg/dL)	18.2	35.92	31.4	17.3

BUN (mg/dL)	85	148	187	86

Lowest creatinine at recovery (mg/dL)	0.92	1.06	1.31	1.6

Cause	Bilateral ureteral obstruction	Urethral obstruction	Bladder outlet obstruction	Endometriosis

Pathology findings	Plasmacytomas	Prostate adenocarcinoma	Benign prostatic hyperplasia	Endometriosis

Management	Percutaneous nephrostomy and chemotherapy	Bladder catheterization	Bladder catheterization	Hysterectomy, hormonal therapy, hemodialysis

DDAVP given	Y	N	N	N/A

Days to lowest creatinine	51	1	5	44

Peak serum potassium (mmol/L)	6.4	7.9	7.4	6.0

pH	7.25	7.12	7.18	7.33

Bicarbonate (mmol/L)	17.1	10.4	10	17

pCO2 (mmHg)	42.7	33.5	20.8	32.8
